# Predictors of weight reduction effectiveness with liraglutide in diabetes mellitus type 2 patients: a retrospective cohort study

**DOI:** 10.1186/s12902-025-02066-0

**Published:** 2025-10-23

**Authors:** Pitsinee Wangpattanamongkol, Worapaka Manosroi

**Affiliations:** 1 Internal Medicine Department, Faculty of Medicine, Chiang Mai University, Chiang Mai, Thailand; 2 Division of Endocrinology, Internal Medicine Department, Faculty of Medicine, Chiang Mai University, 110 Intrawarorot Road Soi 2, Si Phum, Amphoe Mueang, Chiang Mai, 50200 Thailand

**Keywords:** Liraglutide, Weight reduction, Diabetes mellitus type 2, GLP-1 receptor agonist

## Abstract

**Background:**

GLP-1 receptor agonists (GLP-1RAs) are effective for treating type 2 diabetes mellitus (T2DM) and promoting weight loss, but not all patients respond similarly. This study aimed to identify predictors of significant weight reduction at 6 months in T2DM patients treated with liraglutide.

**Materials and methods:**

A retrospective analysis was conducted on 209 T2DM adults prescribed liraglutide at the Internal Medicine Department, Faculty of Medicine, Chiang Mai University between January 1, 2017, and December 31, 2022. Patients who could not tolerate or did not complete titration to 3.0 mg daily were excluded. Demographic, clinical, anthropometric, and biochemical data were obtained from electronic medical records. Logistic regression was used to identify clinical and biochemical factors associated with significant weight reduction (≥ 5% at 6 months), presented as odds ratios (ORs) with 95% confidence intervals (CIs). Statistical significance was set at *p* < 0.05.

**Results:**

Of the 209 patients, 27.3% achieved significant weight reduction. Lower baseline body mass index (BMI) (OR 1.12, 95% CI 1.01–1.25, *p* = 0.036) and greater weight loss at 3 months (OR 2.22, 95% CI 1.67–3.03, *p* < 0.001) were significant predictors of weight reduction at 6 months, after adjusting for age, sex, baseline HbA1c, creatinine, systolic blood pressure, LDL cholesterol, and concomitant medications.

**Conclusion:**

Lower baseline BMI and early weight loss at 3 months predict significant weight reduction with liraglutide therapy. Identifying these simple, accessible predictors can improve patient selection and optimize weight control outcomes.

## Introduction

Over the past decade, the incidence of overweight and obesity has increased significantly worldwide [1]. According to the new definition, obesity is characterized by excessive adiposity, with or without abnormal distribution or function of adipose tissue [2]. It is considered a significant risk factor for metabolic syndrome and type 2 diabetes (T2DM), which can lead to multiple atherosclerotic cardiovascular diseases (ASCVD), increasing rates of mortality and disability [3]. Obesity is a condition that can be managed through various treatment approaches, including behavioral and lifestyle modifications, medication, and bariatric surgery. A weight reduction of at least 5–10% of initial body weight has shown health benefits in reducing risk factors for ASCVD [4].

GLP-1 receptor agonists (GLP-1 RAs) are a class of medications that can help reduce weight and lower blood sugar levels by increasing the insulin response to glucose in the body, slowing gastric emptying, decreasing glucagon secretion, and crossing the blood-brain barrier to stimulate pro-opiomelanocortin (POMC) and other anorexigenic neurons, resulting in early satiety [5]. GLP-1 RAs are recommended for adults with T2DM and overweight/obesity to achieve clinically meaningful weight loss, and are favored in those with established ASCVD due to demonstrated cardiovascular benefit [6]. Patients taking GLP-1 RAs, such as liraglutide, have shown highly variable weight reduction ranging from 3% to 9% of their initial body weight within one year, depending on the dose of the GLP-1 RAs [7]. Approximately 30–60% of study participants using GLP-1 RAs have been reported to achieve significant weight loss (≥ 5% at 6 months), as demonstrated in real-world data and meta-analyses [8, 9].

Regarding predictive factors for significant weight loss with GLP-1 RAs, a phase 2 study using liraglutide 3.0 mg in patients with obesity showed that early responders to weight reduction were more likely to achieve intermediate-term results (more than 5% weight loss at one year of liraglutide use) [7]. Another study revealed no significant association between sex, age, diabetes duration, or initial body mass index (BMI) and the rate of body weight loss after the use of GLP-1 RAs [10].

A clearer understanding of the factors that predict significant weight reduction in GLP-1 RAs users can improve clinical application, maximizing benefits, minimizing side effects, and providing an efficient treatment approach for patients with T2DM. This study aimed to identify clinical and biochemical factors associated with significant weight loss (> 5% in 6 months) in T2DM patients using liraglutide 3.0 mg once daily, helping physicians more effectively select patients who are most likely to benefit from this treatment.

## Materials and methods

This is a retrospective cohort study that included adults aged 18 years and older with T2DM who were first prescribed liraglutide at the Internal Medicine Department, Faculty of Medicine, Chiang Mai University, between January 1, 2017, and December 31, 2022. The study followed the principles outlined in the Declaration of Helsinki. It was approved by the Institutional Review Board of the Faculty of Medicine, Chiang Mai University (MED 2566-0043), which granted an exemption from the informed consent requirement due to the retrospective design of the research.

At our center, liraglutide treatment is initiated at 0.6 mg once daily and titrated weekly in 0.6 mg increments up to the target dose of 3.0 mg daily, as tolerated. Patients unable to tolerate escalation to 3.0 mg or who discontinued liraglutide within the 6-month follow-up were excluded from the study. Other exclusion criteria included patients who were diagnosed with type 1 diabetes mellitus, had a recent history of steroid treatment or herbal drug use, were diagnosed with heart failure (defined by an ejection fraction of less than 40% as determined by echocardiogram), were pregnant, had end-stage renal disease requiring peritoneal dialysis or hemodialysis, had undergone bariatric surgery, or used other weight-reduction agents. Additionally, patients with incomplete data on physical examinations and laboratory test results were excluded. The follow-up period was defined as the interval from the date of starting liraglutide to the points of interest at 3 and 6 months. Significant weight reduction was defined as a ≥ 5% weight loss from the initial body weight within 6 months.

Data were obtained from medical records, which included demographic information (e.g., age, gender), medical history (including comorbidities such as hypertension, dyslipidemia, chronic renal failure, metabolic dysfunction-associated steatotic liver disease (MASLD), ischemic heart disease, cerebrovascular disease), and complications of T2DM (diabetic retinopathy, diabetic nephropathy, hypoglycemia, hyperglycemic crisis). Histories of alcohol consumption and tobacco use were also recorded. Additionally, data on current medication use were collected, including types of diabetes medications, lipid-lowering drugs, antihypertensive agents, and side effects of liraglutide (such as nausea and vomiting). Anthropometric measurements, including weight, height, and BMI, as well as systolic and diastolic blood pressure, were collected from patients’ medical records. Biochemical parameters, including HbA1c, fasting blood glucose, LDL cholesterol, creatinine levels, and estimated glomerular filtration rate (eGFR), were also retrieved from the medical files at baseline, 1 month, 3 months, and 6 months.

### Statistical analysis

The data were analyzed using the STATA program, version 17.0. Statistical significance was considered when the p-value was less than 0.05 (two-tailed). For categorical variables, counts and percentages were reported, while for continuous variables with a normal distribution, means and standard deviations (SD) were provided. For non-normally distributed continuous variables, medians and interquartile ranges (IQR) were presented. For inferential statistics, chi-squared or Fisher’s exact test was used for categorical data. Continuous data were analyzed using the independent t-test for normally distributed data or the Mann–Whitney U test for non-normally distributed data. Multivariable logistic regression analyses were performed to identify predictive factors for ≥ 5% weight loss at 6 months, with results reported as odds ratios (ORs) along with 95% confidence intervals (CIs). Regarding sample size calculation, we prespecified a two-sided α = 0.05 (95% confidence) and 80% power. Given the plan to incorporate at least 12 predictors, and assuming that 60% of patients would have significant weight loss during the follow-up period and about 5% would have incomplete data, a minimum of 200 patients was required to accurately identify the significant predictors.

## Results

A total of 209 T2DM patients were included in the study and were categorized into those who experienced significant weight loss (27.3%, *n* = 57) and those who did not experience significant weight loss (72.7%, *n* = 152). The number of females (*n* = 105, 50.2%) and males (*n* = 104, 49.8%) was comparable. The median age was 56 years old (IQR 44–68). The mean body weight at baseline was 88.4 kg (IQR 66–110.8 kg). The mean BMI was 33.1 kg/m^2^ (IQR 26.3–39.8). There were no statistically significant differences between the two groups regarding demographic characteristics, including sex, age, baseline body weight, BMI, and baseline systolic and diastolic blood pressure. Dyslipidemia was numerically less frequent in the significant weight-loss group (66.7% vs. 79.6%; *p* = 0.051); however, this difference did not reach statistical significance. Additionally, there were statistically significant differences in the use of metformin, angiotensin-converting enzyme inhibitors (ACEIs)/angiotensin receptor blockers (ARBs), and clopidogrel, while no significant differences were observed for other comorbidities. The demographic data are as shown in Tables 1 and 2.

**Table 1:** Baseline demographic data

Characteristic	Total case	Non-significant weight loss (< 5%)	Significant weight loss (≥ 5%)	*p*-value
Male, *n*(%)	104 (49.8)	73 (48.0)	31 (54.4)	0.413
Age (years), mean ± SD	56.2 ± 12.5	56.0 ± 12.1	56.9 ± 13.7	0.621
Baseline body weight (kg), mean ± SD	88.5 ± 22.5	88.0 ± 22.6	89.7 ± 22.5	0.645
Baseline BMI (kg/m^2^), mean ± SD	33.0 ± 6.7	32.9 ± 6.7	33.3 ± 6.7	0.689
Baseline SBP	133.1 ± 16.0	133.2 ± 16.2	132.8 ± 15.5	0.894
Baseline DBP	76.5 ± 10.9	76.6 ± 10.7	76.1 ± 11.5	0.739
Underlying disease				
Dyslipidemia, *n*(%)	159 (76.1)	121 (79.6)	38 (66.7)	0.051
Hypertension, *n*(%)	161 (77.0)	122 (80.3)	39 (68.4)	0.070
Ischemic heart disease, *n*(%)	36 (17.2)	22 (14.5)	14 (24.6)	0.085
Peripheral arterial disease, *n*(%)	2 (1.0)	2 (1.3)	0 (0)	0.999
Chronic kidney disease, *n*(%)	36 (17.2)	25 (16.5)	11 (19.3)	0.627
MASLD, *n*(%)	15 (7.2)	12 (7.9)	3 (5.3)	0.764
Stroke, *n*(%)	7 (3.4)	4 (2.6)	3 (5.3)	0.394
OSA, *n*(%)	27 (12.9)	21 (13.8)	6 (10.5)	0.528
Obesity, *n*(%)	46 (23.4)	35 (23.0)	14 (24.6)	0.816
Diabetes complications				
Diabetic retinopathy, *n*(%)	27 (12.9)	20 (13.2)	7 (12.3)	0.866
Diabetic nephropathy, *n*(%)	44 (21.1)	32 (21.1)	12 (21.1)	0.999
Hyperglycemic crisis, *n*(%)	1 (0.5)	2 (0.7)	0 (0)	0.999
Hypoglycemia, *n*(%)	7 (3.4)	6 (4.0)	1 (1.8)	0.677
Lifestyle				
Smoking, n(%)	11 (5.3)	8 (5.3)	3 (5.3)	0.439
Alcohol drinking, n(%)	21 (10.1)	17 (11.2)	4 (7.1)	0.127

*BMI* Body mass index, *SBP* Systolic blood pressure, *DBP* Diastolic blood picture, *ACS* Acute coronary syndrome, *CCS* Chronic coronary syndrome, *OSA* Obstructive sleep apnea, *MASLD* Metabolic dysfunction-associated steatotic liver disease

**Table 2:** Baseline medications and biochemical data

Characteristic	Total case	Non-significant weight loss (< 5%)	Significant weight loss (≥ 5%)	*p*-value
Medication				
Antidiabetic drug, *n*(%)				
● Metformin ● Sulfonylurea ● Pioglitazone ● DPP4 inhibitor ● SGLT2 inhibitor ● Alpha glucosidase inhibitor ● Insulin	165 (79.0) 76 (36.4) 48 (23.0) 42 (20.1) 102 (48.8) 3 (1.4) 86 (41.1)	127 (83.6) 61 (40.1) 40 (26.3) 27 (17.8) 72 (47.4) 2 (1.3) 66 (43.4)	38 (66.7) 15 (26.3) 8 (14.0) 15 (26.3) 30 (52.6) 1 (1.8) 20 (35.1)	0.008 0.064 0.060 0.169 0.498 0.999 0.276
Antihypertensive, *n*(%)				
● ACEI/ARB ● CCB ● Diuretics ● Beta-blocker ● Alpha-blocker ● Hydralazine ● Methyldopa	140 (67.0) 101 (48.3) 60 (28.7) 71 (34.0) 9 (4.3) 6 (2.9) 2 (1.0)	109 (71.7) 73 (48.0) 41 (27.0) 52 (34.2) 8 (5.3) 5 (3.3) 1 (0.7)	31 (54.4) 28 (43.1) 19 (33.3) 19 (33.3) 1 (1.8) 1 (1.8) 1 (1.8)	0.018 0.888 0.365 0.905 0.449 0.999 0.472
Lipid lowering agent, *n*(%)				
● Statin ● Bile acid sequestrant ● Fibrate and derivatives ● Ezetimibe ● Niacin ● Omega-3	182 (87.1) 0 (0) 6 (2.9) 47 (22.5) 0 (0) 3 (1.4)	134 (88.2) 0 (0) 6 (4.0) 31 (20.4) 0 (0) 1 (0.7)	48 (84.2) 0 (0) 0 (0) 16 (28.1) 0 (0) 2 (3.5)	0.449 - 0.192 0.237 - 0.181
Anti-platelet, *n*(%)				
● Aspirin ● Clopidogrel ● Ticagrelor ● Prasugrel ● Cilostazol	80 (38.2) 15 (7.2) 7 (0.4) 2 (0.9) 1 (0.4)	57 (37.5) 5 (3.3) 0 (0) 1 (0.6) 1 (0.6)	23 (40.3) 10 (17.5) 1 (1.8) 1 (1.8) 0 (0)	0.706 0.001 0.273 0.472 0.999
Biochemical data				
FBS, mg/dL (mean ± SD)	150.8 ± 52.5	152.4 ± 52.7	146.7 ± 52.1	0.489
HbA1c, % (mean ± SD)	8.1 ± 1.7	8.2 ± 1.8	7.9 ± 1.6	0.441
LDL, mg/dL (mean ± SD)	92.1 ± 31.6	91.4 ± 2.6	94.3 ± 4.3	0.555
HDL, mg/dL (mean ± SD)	46.9 ± 14.8	47.1 ± 14.9	46.9 ± 14.9	0.954
Cr, mg/dL (mean ± SD)	1.1 ± 0.4	0.9 ± 0.3	1.1 ± 0.4	0.111

*FBS* Fasting blood glucose, *Cr* Creatinine, *ACEIs* angiotensin-converting enzyme inhibitors, *ARBs* angiotensin receptor blockers, *CCB* Calcium channel blocker, *LDL* Low-density lipoprotein, *HDL* High-density lipoprotein

### Predictors for significant weight loss

In a multivariable analysis of predictors for significant weight loss at 6 months following liraglutide use, a lower baseline BMI was associated with a higher likelihood of achieving significant weight loss (OR 1.12, 95% CI 1.01–1.25, *p* = 0.036). Weight loss at 3 months was associated with significant weight loss at 6 months (OR 2.22, 95% CI 1.67–3.03, *p* = 0.0001). No statistically significant associations were found for age, sex, baseline creatinine, baseline HbA1c, changes in HbA1c during treatment, baseline systolic blood pressure, baseline LDL, or other medications. Data are as shown in Table 3.

**Table 3:** Multivariable analysis of predictors of significant weight reduction at 6 months after the use of liraglutide

Demographic data	aOR	95% CI	*p*-value
Age	1.01	0.96–1.06	0.708
Male	1.67	0.48–5.77	0.421
Baseline creatinine	1.95	0.29–13.02	0.491
Lower baseline BMI	1.12	1.01–1.25	0.036
Baseline HbA1C	0.97	0.66–1.42	0.868
Baseline SBP	0.99	0.96–1.03	0.883
Baseline LDL	1.01	0.99–1.03	0.244
No Sulfonylurea	2.08	0.59–7.34	0.254
No Metformin	2.08	0.46–9.32	0.338
No Insulin	2.29	0.59–8.83	0.228
No SGLT2 inhibitor	1.16	0.36–3.76	0.797
No ACEI/ARB	2.34	0.72–7.62	0.158
Weight loss at 3 months	2.22	1.67–3.03	0.001
HbA1C changes at 3 months	0.96	0.60–1.51	0.852

*aOR* adjusted odds ratio, *BMI* Body mass index, *SBP* systolic blood pressure, *LDL* Low-density lipoprotein, *SGLT2* Sodium-glucose cotransporter 2, *ACEI/ARB* Angiotensin converting enzyme/angiotensin receptor blocker

## Discussion

This retrospective cohort study emphasizes that certain clinical and biochemical markers may predict short-term weight loss at 6 months in T2DM patients treated with liraglutide. Specifically, a lower baseline BMI and early weight loss at 3 months were associated with greater weight loss at 6 months. Identifying these patterns can help clinicians select patients who are most likely to benefit from liraglutide, improving treatment decisions. By recognizing early predictors, our study aims to support personalized liraglutide use, ensuring that patients most likely to respond are prioritized. This approach promotes cost-effective, efficient care by allowing early identification of ‘responders’ and minimizing prolonged ineffective treatment for ‘non-responders’.

Consistent with prior studies, early weight loss with GLP-1 RAs (e.g., ≥ 5% at 1 month or ≥ 4% by ~ 16 weeks) predicts subsequent (6–12-month) efficacy and helps identify patients likely to achieve significant weight reduction with liraglutide [11, 12]. An early response suggests heightened sensitivity to the drug’s effects on appetite suppression, satiety, and energy expenditure. It may also reflect better adherence to lifestyle modifications, such as improved diet, increased physical activity, and consistent use of GLP-1 RAs.

Lower baseline BMI was associated with a significantly higher likelihood of achieving weight loss. Individuals with higher BMI often have greater fat mass and a higher degree of insulin resistance and metabolic adaptation, which can reduce the effectiveness of weight-loss medications like liraglutide [13]. In these individuals, achieving the same level of satiety or energy balance may require more time or higher doses. Additionally, factors such as leptin resistance or impaired satiety signaling may diminish liraglutide’s appetite-suppressing effects. In contrast, individuals with lower BMI typically have a higher proportion of lean body mass and may experience more pronounced weight loss compared to those with higher fat mass [14]. Although lower baseline BMI was statistically associated with achieving ≥ 5% weight loss at 6 months (aOR 1.12 per 1 kg/m² lower BMI), the per-unit effect is modest. In practice, early 3-month weight change—which showed a larger adjusted effect—may be a more actionable marker of response. Accordingly, baseline BMI should be interpreted as a supportive factor within shared decision-making rather than a gatekeeper for therapy. Previous studies have reported that lower baseline HbA1c is a predictor of weight loss in GLP-1 RAs users [15]. However, in the present study, baseline HbA1c showed no significant association with weight loss.

Although our multivariable analysis did not identify concomitant diabetes medications as statistically significant predictors of weight loss, it is notable that patients in the significant weight loss group tended to use more sodium-glucose cotransporter-2 inhibitors (SGLT2i) and fewer sulfonylureas, pioglitazone, and insulin. Since SGLT2i are known to promote modest weight reduction, while sulfonylureas, pioglitazone, and insulin are generally associated with weight gain, these treatment differences may have influenced weight outcomes despite not reaching statistical significance. This observation highlights the importance of considering background therapy when interpreting predictors of weight loss in patients treated with GLP-1 RAs. To address the possibility of confounding by concomitant therapy, we reviewed medication histories and confirmed that all agents with potential effects on body weight, such as insulin, sulfonylureas, pioglitazone, and SGLT2 inhibitors, had been prescribed prior to liraglutide initiation. None of these medications were started concurrently with liraglutide, reducing the likelihood that simultaneous initiation of other therapies confounded the observed weight outcomes.

Our study included 209 adults with type 2 diabetes mellitus, used a predefined and clinically meaningful endpoint (≥ 5% weight reduction at 6 months), and applied multivariable analyses to adjust for measured confounders. Nevertheless, as a retrospective observational analysis, causal inference is limited. The sample size is sufficient for statistical analysis, allowing the detection of significant predictors and trends. The study also focuses on a specific and clinically meaningful outcome—≥5% weight reduction within 6 months—a widely accepted threshold for clinically significant weight loss. Additionally, the use of multivariable logistic regression analysis, adjusting for multiple confounders, enhances the accuracy of the results.

Several limitations should be considered. The retrospective design and reliance on patient records may have led to incomplete data collection, particularly regarding lifestyle factors such as dietary habits, daily calorie intake, physical activity and behavioral support as well as adherence to liraglutide and concomitant therapies which could influence the results. Due to financial constraints, blood tests to measure hormone levels (e.g., insulin, ghrelin, leptin, GLP-1, adiponectin, GIP, and C-peptide) were not performed, unlike previous studies [10]. Additionally, the degree of insulin resistance (homeostatic model assessment for insulin resistance: HOMA-IR) and pancreatic beta-cell function using a meal tolerance test were not assessed, which could help identify patients best suited for GLP-1 RAs treatment. Because waist circumference and waist-to-hip ratio were not consistently available in the medical records, we could not assess abdominal obesity or its relationship to insulin resistance (e.g., HOMA-IR) and liraglutide response; future prospective studies should include standardized measures of central adiposity. In addition, the 6-month follow-up captures only short-term outcomes; we could not assess the durability of weight loss, potential weight regain, or longer-term cardiometabolic endpoints. Future prospective studies with longer follow-up (≥ 3–5 years) are needed to evaluate sustained effectiveness. Lastly, as the study population is predominantly from a single geographic region, ethnicity, and healthcare system, the findings may not be fully generalizable to all T2DM patients globally.

## Conclusion

Lower baseline BMI and greater weight loss at 3 months can help predict short-term weight loss outcomes at 6 months with liraglutide therapy. Identifying simple and accessible predictors based on these parameters will improve patient selection for this treatment, leading to better weight control. Further studies are needed to confirm the causal relationships between these predictors and weight loss outcomes with liraglutide. Developing a predictive model or risk score using these predictors could personalize GLP-1 RAs treatment, enhancing precision medicine and improving patient outcomes.

## Data Availability

The data that support the findings of this study are available upon request from the authors by contacting corresponding author.
